# Chemotherapy in pediatric brain tumor and the challenge of the blood–brain barrier

**DOI:** 10.1002/cam4.6647

**Published:** 2023-11-23

**Authors:** Johid Reza Malik, Anthony T. Podany, Parvez Khan, Christopher L. Shaffer, Jawed A. Siddiqui, Janina Baranowska‐Kortylewicz, Jennifer Le, Courtney V. Fletcher, Sadia Afruz Ether, Sean N. Avedissian

**Affiliations:** ^1^ Antiviral Pharmacology Laboratory College of Pharmacy, University of Nebraska Medical Center Omaha Nebraska USA; ^2^ Pediatric Clinical Pharmacology Program Child Health Research Institute, University of Nebraska Medical Center Omaha Nebraska USA; ^3^ Department of Biochemistry and Molecular Biology University of Nebraska Medical Center Omaha Nebraska USA; ^4^ Department of Pharmaceutical Sciences College of Pharmacy, University of Nebraska Medical Center Omaha Nebraska USA; ^5^ University of California San Diego Skaggs School of Pharmacy and Pharmaceutical Sciences San Diego California USA

**Keywords:** blood–brain barrier, central nervous system, chemotherapy, oncology, pediatric brain tumor

## Abstract

**Background:**

Pediatric brain tumors (PBT) stand as the leading cause of cancer‐related deaths in children. Chemoradiation protocols have improved survival rates, even for non‐resectable tumors. Nonetheless, radiation therapy carries the risk of numerous adverse effects that can have long‐lasting, detrimental effects on the quality of life for survivors. The pursuit of chemotherapeutics that could obviate the need for radiotherapy remains ongoing. Several anti‐tumor agents, including sunitinib, valproic acid, carboplatin, and panobinostat, have shown effectiveness in various malignancies but have not proven effective in treating PBT. The presence of the blood–brain barrier (BBB) plays a pivotal role in maintaining suboptimal concentrations of anti‐cancer drugs in the central nervous system (CNS). Ongoing research aims to modulate the integrity of the BBB to attain clinically effective drug concentrations in the CNS. However, current findings on the interaction of exogenous chemical agents with the BBB remain limited and do not provide a comprehensive explanation for the ineffectiveness of established anti‐cancer drugs in PBT.

**Methods:**

We conducted our search for chemotherapeutic agents associated with the blood–brain barrier (BBB) using the following keywords: Chemotherapy in Cancer, Chemotherapy in Brain Cancer, Chemotherapy in PBT, BBB Inhibition of Drugs into CNS, Suboptimal Concentration of CNS Drugs, PBT Drugs and BBB, and Potential PBT Drugs. We reviewed each relevant article before compiling the information in our manuscript. For the generation of figures, we utilized BioRender software.

**Focus:**

We focused our article search on chemical agents for PBT and subsequently investigated the role of the BBB in this context. Our search criteria included clinical trials, both randomized and non‐randomized studies, preclinical research, review articles, and research papers.

**Finding:**

Our research suggests that, despite the availability of potent chemotherapeutic agents for several types of cancer, the effectiveness of these chemical agents in treating PBT has not been comprehensively explored. Additionally, there is a scarcity of studies examining the role of the BBB in the suboptimal outcomes of PBT treatment, despite the effectiveness of these drugs for other types of tumors.

## 1 INTRODUCTION

Pediatric brain tumors (PBTs) comprise 25% of all childhood cancers,^1^, ^2^ and are one of the leading causes of cancer‐diagnosed death in children.^3^ From surgical removal of PBT followed by radiation therapy and adjuvant chemotherapy to immunotherapy, PBT treatment has substantially improved throughout the years.^1^, ^4^, ^5^ Improvement in gross total resection (GTR) or subtotal resection (STR) of PBT has resulted in the targeted removal of the tumor and enhanced radiological treatment.^6^ For example, proton beam treatment is one promising radiation therapy as it allows targeted dosing at high levels with reduced surrounding tissue damage.^7^ Unfortunately, despite advancements, radiation therapy can cause hearing loss, impaired neurocognition, and alteration in neuroendocrine function, among other adverse events.^8^, ^9^, ^10^ The detrimental effect of radiotherapy in the modulation of BBB integrity was examined in immunocompetent and immunocompromised mice, and it was found that 12‐h irradiation in immunocompetent mice caused alteration of efflux transporter activity compared to immunocompromised mice indicating a role of proinflammatory molecules in BBB structural changes.^11^ Similarly, human clinical trials, in vitro, and in vivo studies have shown leaky BBB caused by irradiation of central nervous system (CNS).^12^, ^13^, ^14^ Cell death augmented by radiotherapy is one of the underlying mechanisms for leakage in the BBB, and one report showed a 15% decline in endothelial cell population post 24‐h irradiation with a 25 Gy dose.^15^ The reactive oxygen species (ROS) production from irradiation can indirectly damage the BBB by inducing apoptosis which starts as early as 4‐h after irradiation, and the effect was observed maximum post 12‐h of irradiation.^16^, ^17^, ^18^ Though several PBTs respond well to radiation, this comes at the cost of potential long‐term neurological consequences, particularly a problem in young children with developing brains.^19^ Immunotherapy of PBTs is emerging as a novel adjuvant monotherapy in the post‐radiotherapy setting, and early clinical trials show overall safety, feasibility, and survival benefit in patients.^20^ While it is still progressing, the adverse effects of immunotherapy are a serious concern, with immune‐related adverse effects showing up as early as 3 months post‐therapy.^21^ Unfortunately, there is limited success with combinations of radiotherapy, neurosurgery, and chemotherapy.^22^, ^23^, ^24^, ^25^ Considering the overall side effects of the current therapy to treat PBTs’,^26^ chemotherapy becomes the treatment choice for controlling the residual and micrometastatic tumors that cannot be removed by surgery.^27^ However, BBB, being important for regulating which molecules can pass from the blood into the brain, can hinder drug penetration leading to suboptimal drug concentrations in the CNS.^28^, ^29^

In 1979, chemotherapy became part of standard‐of‐care therapy as an adjuvant to surgery and radiation in PBTs. In patients with medulloblastoma (MB) with or without metastacies, the addition of chemotherapy as an adjuvant significantly improved the event‐free survival rate up to 86% ± 9%.^1^ Several combinations of anti‐tumor drugs have been optimized to a range of 4–9 treatment cycles, depending on the risk assessment of the disease.^30^ Interestingly, in many cancers, including MB, it has been observed that children tolerate chemotherapy better than adults.^31^ While the exact mechanism of this tolerance is unknown, it is believed to be a combination of altered hepatic metabolism, diminished resistance to treatment regimens, and fewer concurrent disease states as compared to adult patients.^32^ Nonetheless, chemotherapy causes significant adverse effects, such as post‐treatment pancytopenia, encephalopathy, ataxia, and motor weakness, among many other undesirable effects.^33^ A systemic approach for chemotherapy involving the evaluation of molecular basis/checkpoints, the related epigenetics, and the genomic level study has increased efficacy and lowered the risk for toxicity in patients.^34^, ^35^, ^36^ Through the HIT‐2000 trial, it was established that a systemic drug regimen in combination with intraventricular methotrexate is better than craniospinal irradiation in children >4 years of age for the treatment of nonmetastatic MB.^37^ Even for treating high‐risk PBTs in children >3 years of age, chemotherapy can be applied to the radiologically inaccessible residual tumor. In a randomized clinical trial of 261 patients with group 3 MB subtype, carboplatin use during radiotherapy increased survival rate from 54% to 73%.^38^

Despite the remarkable success of chemotherapy in PBTs, improvements in patient survival remain a significant concern and challenge, and tumor subtype‐associated differential success under chemotherapeutic treatment needs to be comprehensively explored.^27^ This premise requires understanding and characterizing anti‐tumor drug pharmacology in all histologically and clinically diverse PBT subtypes. Within the scope of this review, we discuss the most common PBTs and their chemotherapies with an emphasis on the BBB's role in drug delivery and the efficacy of treatment.

## 2 PBT AND THE BLOOD–BRAIN BARRIER

Among the different pediatric brain cancers, MB is most prevalent, representing approximately 20% of all brain‐related cancers in children.^39^, ^40^ Cerebellum‐originated MBs have been found among all ages, but children with a median age of 5 years show the highest incidence of MBs.^41^ MBs are the first PBT to have its own Medulloblastoma Advanced Genomics International Consortium (MAGIC), which has provided insight into the molecular basis of MBs, leading to better clinical results.^42^ In 2006, the WHO classified the subgroup of cancers within MBs into four subtypes: Wingless/Integrated (WNT)‐activated, Sonic Hedgehog (SHH)‐activated, group 3 and group 4 with distinct genetic makeup which is essential for clinical differentiation.^43^, ^44^

Diffuse intrinsic pontine glioma (DIPG), a high‐grade glioma (HGG), is an aggressive PBT with poor survival that accounts for ~75% of brain stem tumors in children.^39^ The WHO classification for pediatric high‐grade gliomas (pHGGs) indicates diffused astrocytoma as grade II, grade III for anaplastic astrocytomas, and grade IV for glioblastomas.^45^ The histone mutations HIST1H3B, H3F3A, and G34 are considered a predominant subgroup in pHGGs. Detailed molecular characterization and epigenetics of pHGGs have been carried out by various researchers.^46^, ^47^, ^48^, ^49^ Although there has been progress in understanding the subgroups of HGG, distinguishing it from the adult form of HGG, improvement for pediatric glioma is needed as mortality remains high at 43% for children up to 14 years old with PBT.^50^

Ependymoma is a relatively less common PBT (https://tumourclassification.iarc.who.int/login?redirecturl=%2Fchapters%2F45), and constitutes around 10% of the total childhood brain tumors reported.^51^ Interestingly, there seems to be a male predominance among ependymoma cases in patients <5 years of age.^51^, ^52^ A literature review by Sun and colleagues evaluated sex discrepancy in brain tumor biology.^53^ They found that brain tumors occur more frequently in males compared to females regardless of age, tumor histology, or region of the world. They hypothesized that sexually dimorphic mechanisms might control tumor cell biology, as well as immune and brain microenvironmental responses.^53^ According to the WHO classification, the subgroup of ependymoma includes subependymoma and maxillary ependymoma (grade I), classic (grade II), and anaplastic (grade III).^54^ Given the intrinsic association of the CNS and the BBB in drug pharmacology, designing successful chemotherapy regimens and understanding the PBT‐BBB‐drug axis is crucial for maximizing therapeutic effects.

### 2.1 Blood–brain barrier

**FIGURE 1:**
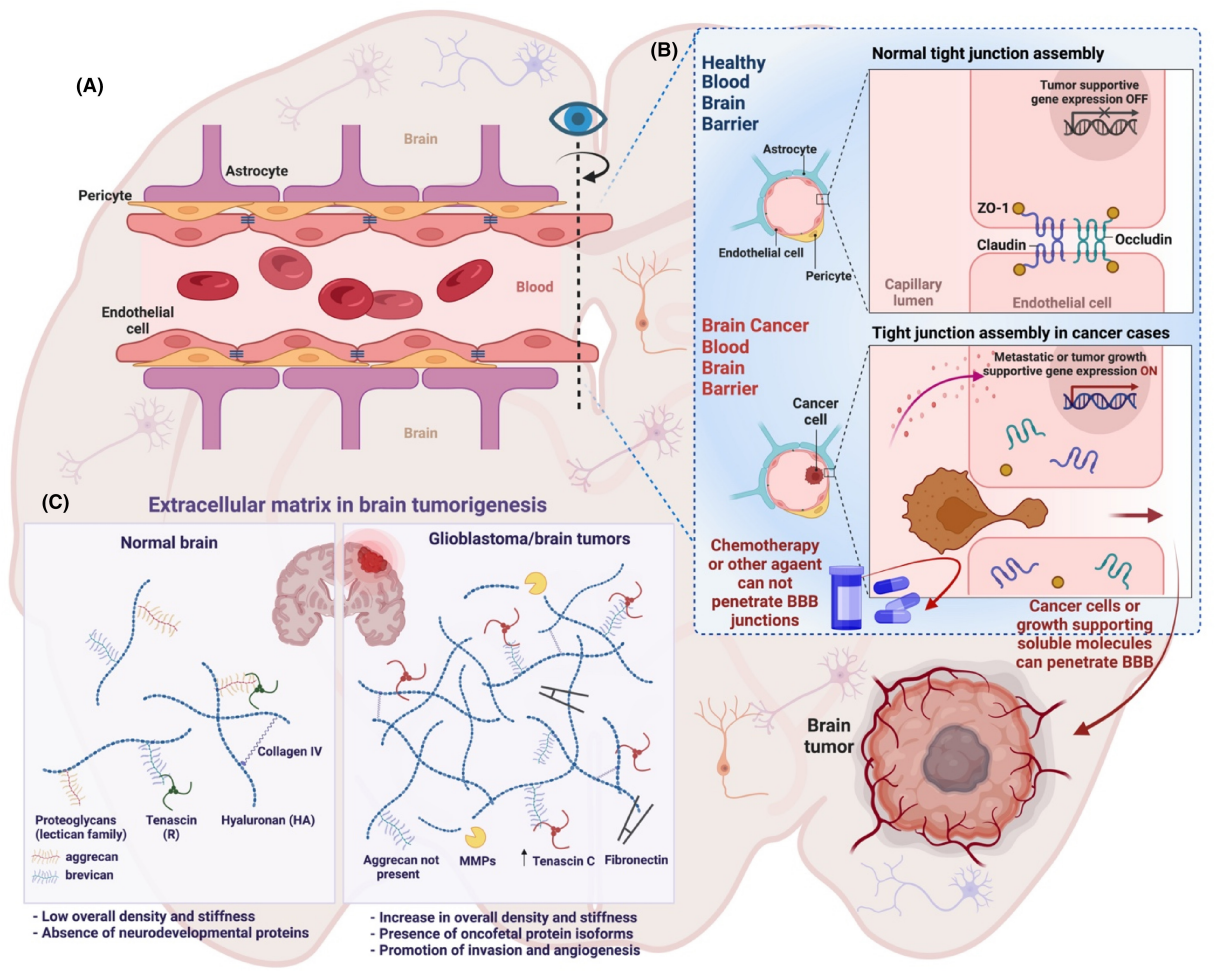
Presentation of BBB interplay with PBT, (A) Constituent cells of BBB and the in vivo environment, (B) enlarged part of CNS depicting BBB in healthy brain and brain tumor with the associated BBB inhibition of drug entry, and (C) molecular‐level comparison of the normal brain with a brain tumor. BBB, blood–brain‐barrier; PBT, pediatric brain tumor; CNS, central nervous system; MMPs, metrix metalloproteases. *Figure was generated utilizing Biorender.com.

The BBB is a protective vascular barrier keeping the brain safe from the detrimental effect of toxins and pathogens.^55^, ^56^, ^57^ The structural component of the BBB primarily includes microvascular brain endothelial cells (MBECs) lining the cerebral blood vessels,^58^ pericytes that share the basement membrane with endothelial cells,^59^ and astrocytes with their tendrils for communication with neighboring cells^60^, ^61^ (Figure 1). Expression of tight junction proteins, namely occludins, claudins, junctional adhesion molecules, and cytoplasmic accessory proteins by MBECs, astrocytes, and pericytes play a pivotal role in barrier formation.^62^, ^63^, ^64^ Despite tight junction formation by peripheral capillary endothelial cells, the TEER (Transepithelial electrical resistance) value observed is 2‐fold less when compared to the BBB, pointing to a bidirectional paracellular transport of molecules across the capillary endothelial cells.^65^, ^66^ The BBB‐associated brain endothelial cells are distinct from capillary endothelial cells and exhibit extensive fenestration and enhanced tightness of intercellular junctions with lower pinocytotic function.^65^, ^67^, ^68^ The unique features of the BBB enable ionic homeostasis and optimal nutrition maintenance in the CNS.^69^ There is passive permeability for essential water‐soluble nutrients across the BBB, while other nutrients engage with specific transporters for nervous tissue requirements.^70^, ^71^ It is important to understand the specific role played by the BBB in PBTs as the BBB can present physiological obstacles for pharmacologic agents used in the treatment of PBTs.

The constituent cells of the BBB express efflux transporters, including ATP‐binding cassette (ABC) proteins P‐glycoprotein (Pgp) and breast cancer resistance protein (BCRP). These efflux transporters, as crucial as they are to BBB regulation, can pump out pharmacologically important molecules from the brain.^72^, ^73^ The critical role of these protein pumps has been shown in knock‐out animals, confirming that many small molecules used as drugs are a substrate for these protein pumps and are excluded by the BBB resulting in lower efficacy of the drugs.^74^, ^75^, ^76^ The integrity of the BBB in the tumor region, also known as blood tumor barrier (BTB), varies based on the type and subtypes of tumor.^77^ Limited studies have shown BBB integrity modulation for different PBTs (Table 1). Midline glioma (DMG) is a subtype of HGGs where the BTB is seen intact, whereas adult glioblastoma has been shown to express a leaky BTB.^77^, ^78^ Likewise, it was demonstrated that there is a substantial difference in the BBB of WNT‐activated and SHH‐activated MBs subtypes.^79^ The establishment of aberrant vascular networks in the WNT‐activated MB impacts paracrine signaling activity, which creates a non‐functioning BBB and allows enhanced chemotherapeutic concentrations as compared to SHH‐activated MB. The heterogeneous alteration in the BBB in adult brain tumor and PBTs and their subtypes affect the permeability, bioavailability, and chemotherapeutic response of potential therapeutic chemoagents.^79^, ^80^ Even though molecular identification and targeted therapy for PBTs have come a long way, better strategies are needed to improve drug penetration and thereby the efficacy of current and future therapeutic agents.

**TABLE 1:** Level of damage to the blood–brain‐barrier integrity in different types of PBT*.

PBT type	PBT subtypes with Intact BBB	PBT subtypes with Mild BBB alteration	PBT subtypes with Leaky BBB	References
Medulloblastoma	1. SHH^67^ 2. Group 4 subtype^216^	3. Group 3 Subtype^216^	4. WNT‐activated medulloblastoma^67^	Phoenix 2016^79^, Mo 2002^240^
Glioma	1.DIPG (DMG)^218^ 2. Peripheral glioma^217^	3. pHGG^218^	4. Late Glioma or core of Glioma^217^	Gao 2018^241^, Wei 2002^242^
Ependymoma*	1. Intracranial ependymoma^219^ 2. Spinal ependymoma^219^ (based on ABC transporter expression)	3. Myxopapillary ependymomas^199^ (based on ABC transporter expression	No data available	Ginguené 2010^214^, Duda‐Szymańska^243^

*Note*: Intact BBB: Healthy and functional BBB with no signs of damage. Mild BBB alteration: Minimum damage to the BBB with little increased porosity. Leaky BBB: BBB with minimum inhibition of molecules across it, a non‐functional BBB.

Abbreviations: ABC transporter, ATP‐binding cassette transporters; BBB, blood–brain‐barrier; DMG, diffuse midline glioma; PBT, pediatric brain tumor; pHGG, pediatric high‐grade glioma; SHH, sonic hedgehog; WNT, wingless/integrated.

* There are inconclusive reports of BBB integrity in pediatric ependymomas. Thus, this is hypothesized based on the expression of ABC transporters.

To effectively treat MB and the other PBTs, a chemotherapeutic agent must be capable of crossing the BBB to obtain optimal CNS concentrations.^79^, ^81^, ^82^ A drug's molecular size plays a critical role in traversing the BBB. Currently, only 5% of the available drug can pass through the BBB, emphasizing the need to explore and modulate both BBB and therapeutic agents.^83^, ^84^, ^85^ To achieve desired CNS concentrations of potential anti‐tumor agents, it is essential to understand the role of the BBB and its modulation for enhanced drug penetration.

## 3 CHEMOTHERAPEUTIC TREATMENT OF PEDIATRIC BRAIN TUMORS (PBTs)

### 3.1 Medulloblastoma (MB)

Chemotherapeutic agents like cisplatin, carboplatin, lomustine, cyclophosphamide, and vincristine are commonly used in MB treatment^1^, ^86^, ^87^, ^88^, ^89^, ^90^ (Table 2). In a pediatric study, the progression‐free survival (PFS) of children with high‐risk MB improved from 65% to 86% and 79% for 3 and 5 years, respectively, in those treated with craniospinal irradiation and vincristine.^88^ In other pediatric clinical trials of maintenance chemotherapy, lomustine, cisplatin, and vincristine were used to inhibit the resurrection of the disease.^87^, ^88^, ^90^, ^91^ Patients 3–10 years of age receiving adjuvant therapy with chemotherapy experienced a 96% 2‐year survival rate compared to a 59% 2‐year survival rate with radiotherapy alone.^90^, ^91^ Furthermore, patients with advanced stages of MB demonstrate greater benefit of adjuvant chemotherapy compared to early stage MB.^88^ Several studies have shown better response to chemotherapy alone for most MB subtypes like desmoplastic, extensive nodular, or classic MB, since it alleviates the use of radiation therapy.^92^, ^93^, ^94^, ^95^, ^96^, ^97^, ^98^ Rutkowski et al demonstrated that histopathology analysis was a strong independent prognostic indicator for 8‐year event‐free survival and overall survival, where de‐escalation of chemotherapy may be appropriate in young children with desmoplastic/nodular and extensive nodularity type of MB histopathology.^92^ In a different study, the standard risk of MB showed an expected overall survival of about 85% in patients with craniospinal irradiation followed by adjuvant chemotherapy.^1^, ^99^, ^100^ However, in high‐risk MB, this regimen has only a 50% cure rate, where intensive treatment with high‐dose chemotherapeutic agents increases the survival from 20% to 40% and 60% to 70%.^101^, ^102^ In this pursuit of chemotherapy in MB, the phase I study of sonidegib (LDE225) on PBT and phase II for relapsed MB exhibited anti‐tumor activity for patients with relapsed Hh MB, but it was not active against non‐Hh MB.^103^

**TABLE 2:** A summary of important anti‐tumor drugs under trial/investigation for PBT treatment*.

Drug/therapy	Condition/disease	Mechanism of action	Clinical/preclinical trials	Observed side effects	References
Cisplatin	MB	DNA crosslinks/adducts formation	Phase III	Anemia, nephrotoxicity, and excitotoxic neuronal death	Packer 2006^1^, ^87^, Packer 1988^90^, Packer 1994^89^, Sirachainan 2018^236^
Carboplatin*	MB	DNA alkylating agent	Phase III	Peripheral neuropathy, Low blood cell count, and abnormal magnesium level	
Lomustine	MB	DNA crosslinks and alkylating agent	Phase III	Myelosuppression, ulcers, nephrotoxicity, and poor appetite	
Cyclophosphamide	MB	DNA crosslinks formation	Phase I	Apoptotic and excitotoxic neuronal death, a decline in neurogenesis, cytokine dysregulation, reduced glutathione/glutathione peroxidase	
Vincristine	MB	Inhibitor of mitosis at metaphase by interaction with tubulin	Early phase I	Neuronal apoptosis, blurred or double vision, and drooping eyelids	
Sunitinib*	MB	Tyrosine kinase inhibitor	Phase I clinical trials	Increased liver enzymes and low levels of thyroid hormones	Rock 2007^104^
Valproic acid*	MB	Enhanced neurotransmission of GABA	Phase II clinical trials	Congenital anomalies, drowsiness, tremors, dysphoria, and thrombocytopenia	Li^111^
Combination of gemcitabine and ribociclib	MB	CDK4/6 inhibitor	Phase I	Hematologic toxicity, CNS exposure	Pribnow 2022^237^
TB‐403	MB	Neutralizing Ab, Neuropilin1‐PIGF signaling inhibitor	Phase I	Vomiting, diarrhea, nausea, fatigue, decreased appetite, and decrease in immune cell count	Saulnier‐Sholler 2022^128^
Panobinostat*	DIPG	Histone deacetylase inhibitor	Preclinical studies on humans and mouse	Thrombocytopenia, Lymphopenia, neutropenia, low calcium, low albumin, low phosphorus, low hemoglobin, and fatigue	Bradner 2010^145^, Grasso 2015^238^
Difluoromethylornithine (DFMO)*	DIPG	Inhibition of the polyamine synthesis pathway	Preclinical trials	Ototoxicity, gastrointestinal upset, proteinuria, and anemia	Wallace 2003^155^, Rea 2004^154^
Temozolomide*	DIPG	Causes reduction in O^6^‐methylguanine methyltransferase (MGMT), required for DNA repair, and this reduction in the enzyme results in an increased level of O^6^‐methylguanine in DNA, leading to a higher cytotoxic effect	Phase III clinical trials	Stomatitis, dyspepsia, hemorrhoids, convulsions, and hemiparesis	Brada 200^168^, Yung 2000^169^, Yung 1999^170^
Bevacizumab*	DIPG	Antagonist of VEGF	Phase III clinical trials	Belching, nephrotic syndrome, hypertensive crisis, leukopenia, and proteinuria	Ferrara 2004^189^ Friedman 2009^188^
5‐azacytidine	Ependymoma	DNA methylation inhibitor	Pilot clinical trial	Febrile neutropenia, bruising, and leukopenia	Sandberg 2019^220^
Pembrolizumab	Ependymoma	Immune checkpoint inhibitor	Phase II	Anemia and hyperglycemia	Blumenthal 2016^239^

Abbreviations: CDK4/6, cyclin‐dependent kinase 4 or 6; CNS, central nervous system; DIPG, diffuse intrinsic pontine glioma; DNA, deoxyriboneuclic acid; GABA, gamma amino‐butyric acid; MB, medulloblastoma; PIGF, placental growth factor; VEGF, vascular endothelial growth factor.

* Drugs denoted with (*) were discussed in this review. This table is not all‐inclusive and only briefly summarizes some potential chemotherapeutic agents.

#### 3.1.1 Sunitinib

The U.S. Food and Drug Administration (FDA) approved sunitinib (SU11248, Sutent), a tyrosine kinase inhibitor used as a multi‐target agent in cancer angiogenesis.^104^

Chemical structure of Sunitinib. https://pubchem.ncbi.nlm.nih.gov/compound/Sunitinib

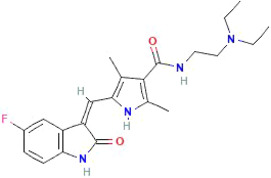

A preclinical report indicated inhibition of MB cancer by sunitinib involving the STAT3‐AKT signaling pathway.^105^ Pharmacologically, the plasma maximum concentration (C_max_) is observed between 6 and 12 h post‐administration, and the bioavailability of oral sunitinib is estimated to be ~50%.^106^, ^107^ Despite proven efficacy in treating renal cell carcinoma,^104^ the success of sunitinib in treating pediatric MB is poor. One potential reason for this could be due to its limited penetration through an intact BBB.^108^ A study by Sobanska and colleagues used a rabbit model to show that the exposure of sunitinib in plasma, aqueous humor, and CSF was different depending on the time of day of drug administration (8 am dose area under the curve [AUC_0−time of last measurable concentration_], CSF: 55.5 ng*h/mL vs. 9 pm dose AUC_0−time of last measurable concentration_, CSF: 66.3 ng*h/mL, respectively).^108^ However, sunitinib penetration through the BBB was reported to be very low (<5%) and comparable in both dosing groups.

To overcome the limitations of the BBB, a study by Szalek and colleagues evaluated the antibiotic ciprofloxacin to modulate the BBB and enhance penetration of sunitinib.^109^ They found that rabbits treated with sunitinib + ciprofloxacin had higher 24‐h CSF exposures (AUC_0–24_ and C_max_) compared to those that only received sunitinib (50.4 vs. 155 ng*h/mL and 4.2 vs. 18 ng/mL).^109^ As clinical outcome data in pediatrics for sunitinib are limited, a phase II clinical multicenter trial conducted by the Children's Oncology Group in 29 children found that sunitinib (as monotherapy) was reasonably well tolerated in children with recurrent ependymoma or high‐grade glioma.^110^ However, the trial was closed at the time of interim analysis as there was no efficacy associated with sunitinib for recurrent PBT. The study concluded that sunitinib lacked anti‐tumor activity as monotherapy.^110^

#### 3.1.2 Valproic acid

Valproic acid (VPA) is a histone deacetylase inhibitor (HDACi) that has shown promise in cancer therapeutics given that histone deacetylase is a key component of epigenetic machinery, and it regulates gene expression through increased histone acetylation, while behaving as oncogenes in some cancers like MB.

Chemical structure of VPA. https://pubchem.ncbi.nlm.nih.gov/compound/ValproicAcid

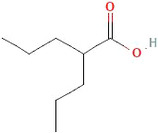

A report by Li and colleagues showed VPA inhibited cancerous growth in the MB cell line.^111^ In a phase I clinical trial of children with brain cancer malignancies, the Children's Oncology Group showed that limiting VPA trough plasma concentrations to 75–100 μg/mL minimized toxicities.^112^ In 2011, the FDA issued warnings for life‐threatening side effects when VPA concentrations exceed 75 μg/mL.^113^ Consequently, balancing the benefit vs. toxicity of VPA is a clinical challenge in the treatment of cancer.

Ionized forms of VPA at a plasma pH of 7.4 render it less permeable through plasma membranes for passive diffusion,^114^, ^115^ and likewise, VPA also has difficulty crossing the BBB. VPA's difficulty in penetrating the CNS is believed to be because VPA acts as a substrate for the ATP‐binding efflux transporter on the BBB.^116^ Given the potential of VPA as an anti‐tumor agent, current studies are exploring the acceleration of VPA influx through BBB. One such study determined that pre‐treatment with *Gastrodia elata* extract substantially improved BBB penetration of VPA due to upregulation of influx transporters, specifically the OATP transporter.^117^ The study found that rats treated with *Gastrodia elata* at oral doses of 1 and 3 g/kg for 5 days increased the BBB AUC penetration ratio from 0.36 to 1.47 and 1.02, respectively.

#### 3.1.3 Carboplatin

Carboplatin is a platinum alkylating agent that covalently binds to DNA. Carboplatin is most commonly used for ovarian cancers. However, carboplatin has shown potential for treatment of other cancers, with studies underway.

Chemical structure of Carboplatin. https://pubchem.ncbi.nlm.nih.gov/compound/Carboplatin

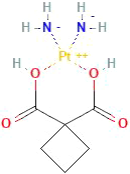

The molecular mechanism of carboplatin is similar to cisplatin but with lower side effects.^118^ In 2021, a randomized controlled trial in 261 children with MB found that carboplatin inclusion during radiotherapy enhanced survival by 19% compared to no carboplatin.^38^ However, improved survival was only observed in the high‐risk group 3 children with MB.^38^

Preclinical in vivo studies have shown enhanced carboplatin penetration through the BBB when co‐administered with RMP‐7, a bradykinin analog.^119^, ^120^ Specifically, Elliott and colleagues showed that intracarotid doses of RMP‐7 from 0.01 to 9 μg/kg significantly increased the permeability of carboplatin into tumor tissue (*F* [6, 144] = 10.92, *p* < 0.001) and surrounding brain tissue (*F* [6, 144] = 9.17, *p* < 0.001) in a dose‐dependent manner.^119^ A study by Matsukado et al^120^ also showed that intracarotid infusions of RMP‐7 increased the transport of carboplatin to tumors by 2.7 fold (*p* < 0.001). This could have clinical implications as they found that the RG2 glioma rats treated with carboplatin and RMP‐7 had increased survival compared to those who only received carboplatin alone (37% vs. 74%).

#### 3.1.4 Vismodegib

Vismodegib is a small molecular inhibitor shown to efficiently inhibit relapse in SHH‐activated MB, where the probability of drug resistance development is high.^121^

Chemical structure of Vismodegib. https://pubchem.ncbi.nlm.nih.gov/compound/Vismodegib

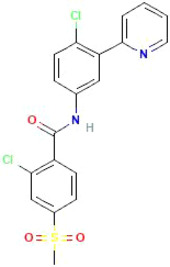

A phase I and phase II clinical trial concluded that vismodegib is an efficient and well‐tolerated drug against pediatric and adult MB, and vismodegib achieves anti‐MB activity by inhibiting the SHH signaling pathway.^122^ This trial reported an objective response rate of 37% for vismodegib but the drug showed no response (0% response rate) in a non‐SHH type MB. Currently, there are limited data on vismodegib's ability to cross the BBB. A recent study published in early 2023 by Tylawsky and colleagues utilized a fucoidan‐encapsulated vismodegib strategy to improve drug delivery across the BBB, and decrease the adverse effect of growth plate fusion observed at clinically effective doses.^123^ They found that in their animal model, fucoidan‐based nanoparticles encapsulating delivery of vismodegib exhibited good efficacy, reduced bone toxicity, and increased drug exposure to healthy brain tissue. This is especially significant to pediatric patients as growth plate fusion can stunt a child's growth potential. Overall, these findings demonstrate a potent strategy for targeted delivery that overcomes the BBB to achieve increased selective tumor penetration and has therapeutic implications for drug delivery to other diseases in the CNS.

#### 3.1.5 TB403

TB‐403, a humanized recombinant IgG1 monoclonal antibody with high affinity to the receptor (Neuropilin‐1) of the placental growth factor (PIGF), inhibits PIGF‐associated stimulation by blocking the PIGF‐neuropilin‐1 ligand–receptor interaction in capillary endothelial cells.^124^ TB‐403 can also interact and have an inhibitory effect with vascular endothelial growth factor receptor 1 (VEGFR1).^124^ PIGF is expressed in MB PBT, produced by the cerebellar stroma via the SHH ligand.^125^ Moreover, PIGF and neuropilin‐1 (Nrp1) signaling play an important role in the growth and spread of MB.^124^ In murine models with human MB xenograft and mimicking clinical symptoms, TB‐403 inhibited primary tumor growth and spinal metastasis by interfering with PIGF and neuropilin‐1 binding.^126^ This preclinical study recorded that in the presence of TB‐403, the mean mouse survival increased from 40 to >55 days. Regarding clinical data, a phase I dose escalation study of TB403 found that the most commonly observed treatment‐emergent adverse events were fatigue, constipation, pyrexia, and dyspnea.^127^ Available data also suggest that the VEGF pro‐angiogenic signaling pathway inhibitors may increase plasma levels of pro‐angiogenic factors such as PIGF, a determinant of drug‐induced resistance to therapy.^124^ The phase I trial of TB‐403 in relapsed MB, neuroblastoma, Ewing Sarcoma, and alveolar Rhabdomyosarcoma indicated its good tolerance in the small population of heavily pretreated advanced solid tumor patients. In this trial, 15 subjects were given 4 dose levels (20, 50, 100, and 175 mg/kg), and the treatment caused a total of 75 adverse events (AEs) in 10 out of 15, but no fatal adverse events were observed during the project. However, serious adverse events were recorded in 3 out of 15 patients treated. The results of the study did not show any conclusive therapeutic response, with 63% of the relapsed MB patients experiencing stable disease conditions for 100 days.^128^ TB‐403 does not require BBB penetration as it exerts its inhibitory effect on ligand–receptor blocking. Nevertheless, investigation for the on‐site effect of the antibody on the distal part of the brain by examining the BBB penetrating capability could improve the therapeutic future of TB403.

### 3.2 Diffuse intrinsic pontine glioma (DIPG)

DIPG is a high‐grade pediatric glioma, a malignant brainstem tumor, with a median survival of <1 year, while less than 10% of patients reported having overall survival >2 years.^129^ The tumor's location makes it difficult for complete resection.^130^ In children, DIPG accounts for 80% of brainstem tumors.^131^, ^132^ Histological analysis reveals a close similarity between grade III anaplastic astrocytomas and grade IV glioblastoma.^45^ In 50% of the patients, clinical symptoms include cranial nerve palsies, long tract signs, cerebellar ataxia, and dysmetria.^133^, ^134^ In DIPG, the most affected nerves are cranial nerves VI and VII, and these nerves' altered function is a symptomatic characteristic of DIPG.^133^ Common and standard practices for DIPGs comprise a 54–59 Gy dose of fractionated radiation because of the interior location of the tumor.^135^ Early approaches of monotherapy or combined chemotherapies have failed to work against DIPG cancer efficiently.^136^, ^137^, ^138^ It is believed that the oncogenic drivers for the DIPGs are the mutations in the histone protein, either by somatic‐like mutations in H3K27M or H3K27 trimethylation.^47^ Since the discoveries of histone protein mutations responsible for this disease, several molecular inhibitors for histone deacetylase and demethylase have been evaluated for potential therapeutic application.^139^, ^140^, ^141^ For DIPG, chemotherapeutic options are inefficient because of the intact BBB, which restricts the delivery of drugs to DIPG tumors.^142^ However, there is an indication of SHH‐mediated signaling of lower BBB permeability in DIPG.^143^ Regardless of the recent advances in identifying a target and specific drug, drug delivery failure across the BBB remains a significant challenge, and drug effectiveness against other tumors fails to inhibit DIPG.^144^

#### 3.2.1 Panobinostat

**FIGURE 2:**
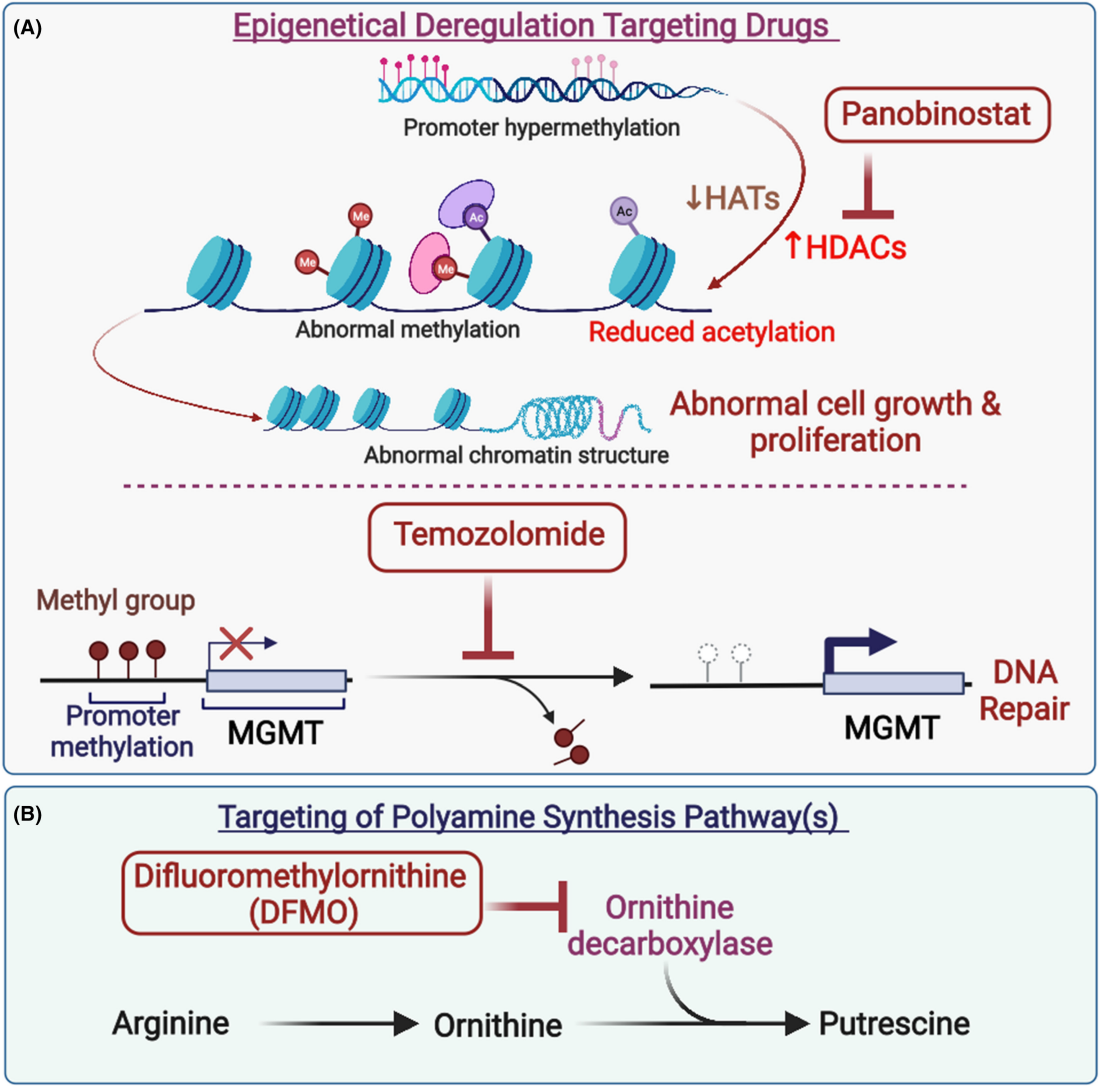
Major pathways or targets of (A) epigenetic drugs panobinostat (upper panel) and temozolomide (lower panel), and (B) polyamine synthesis targeting drug difluoromethylornithine. HATs, histone acetyltransferase; HDACs, histone deacetylase; MGMT, O6‐methylguanine‐DNA methyltransferase; DFMO, difluoromethylornithine. *Figure was generated utilizing Biorender.com.

Panobinostat is a histone deacetylase inhibitor that acts as a potent inhibitor of DIPG, and the epigenetic dysregulation is depicted in Figure 2A. It was first identified by Grasso, C.S et al. while performing chemical screenings against DIPG.^139^, ^145^

Chemical structure of Panobinostat. https://pubchem.ncbi.nlm.nih.gov/compound/Panobinostat

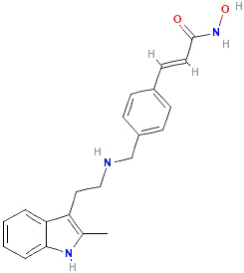

In the presence of panobinostat, there was a significant decrease in proliferation via the upregulation of genes MKI67 and CCND1.^139^ The tumor inhibitory effect of panobinostat was validated in DIPG mouse and H3.3K27M orthotopic xenograft models.^146^, ^147^ It was shown that panobinostat improved the epigenetic effect on mesenchymal stem cells tumor necrosis factor‐related apoptosis‐inducing ligand (MSCs TRIAL), resulting in tumor growth arrest and an increase in overall survival by 5.5 days compared to control group.^148^, ^149^ In vitro analysis based on western blotting showed dose‐dependent enhancement in H3 acetylation and H3K27 trimethylation in panobinostat‐treated cells expressing H3.3K27M mutation.^139^ RNA‐seq data from the study also supported the normalization of the K27M gene while decreasing oncogenic target gene expression in panobinostat‐treated cells.^139^ Further, preclinical studies on human cells and mouse DIPG have confirmed panobinostat as an efficient chemical agent against DIPG.^146^ Preclinical studies for this inhibitor alone or in combination with other compounds have shown a better survival rate in a synergistic approach in several studies.^139^, ^150^

Although the exact reason for the inefficient outcome of this drug when used alone is still not completely understood, poor BBB penetration has been proposed as a significant contributor to the diminished potency of panobinostat.^151^, ^152^ An in vitro study by Hennika and colleagues tested this theory by administering mice with different regimens of panobinostat. They found that extended daily consecutive treatment in both genetic and orthotopic xenograft models was required to get adequate exposure in the brain, but this came with significant toxicity.^146^ Efforts are being made to enhance the BBB penetration of panobinostat to achieve desired CNS concentrations. It was found that convection‐enhanced delivery (CED) combined with positron emission tomography (PET) might have utility in increasing BBB penetration.^153^ A study by Tosi and colleagues utilized both CED and PET to modulate the CED infusions of panobinostat to ensure saturation of the tumor by drug.^153^ They concluded that personalized image‐guided drug delivery might be useful in potentiating CED‐based treatment algorithms to support clinical translation of panobinostat for improvement in survival rates in pediatric diffuse midline glioma.

#### 3.2.2 Difluoromethylornithine (DFMO)

DFMO is a small molecule that irreversibly inhibits the polyamine synthesis pathway, inhibiting cell proliferation^154^, ^155^, illustrated in Figure 2B.

Chemical structure of DFMO. https://pubchem.ncbi.nlm.nih.gov/compound/Difluoromethylornithine

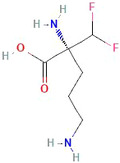

Polyamines are one of the major substrates in intracellular biosynthesis and catabolic pathways and are thus tightly regulated.^156^, ^157^ DFMO inhibits the activity of ornithine decarboxylase1 (ODC1), which is required for the decarboxylation of ornithine into polyamine putrescine.^158^, ^159^, ^160^ ODC1, which has different activity levels in response to growth stimuli, is found to be upregulated in cancer.^161^ Though its effect on adult cancers is underwhelming,^162^, ^163^ the use of DFMO in childhood cancer has potential given its activity in neuroblastoma cell lines.^164^, ^165^ Recent data demonstrated hyperactivity of the polyamine pathway in DIPG preclinical in vitro and in vivo models.^166^ Briefly, this study showed that DFMO, combined with polyamine transport inhibitor AMXT 1501, significantly increased the survival rate of mice to 160 days compared to 60 days for control group in the orthotopic DIPG model. Brain polyamines have also been shown to break down the integrity of BBB. Interestingly, given DFMO's inhibition of the synthesis of polyamines, it has been shown to decrease the postischemic breakdown of the BBB.^167^ Consequently, BBB resistance to other synergistic molecules is risky in DIPG treatment.^167^

#### 3.2.3 Temozolomide

Temozolomide is an orally bioavailable agent and has proven function against high‐grade gliomas and has exhibited a better clinical effect than procarbazine (not discussed in this review), another potent tumor inhibitor.^168^, ^169^, ^170^

Chemical structure of Temozolomide. https://pubchem.ncbi.nlm.nih.gov/compound/Temozolomide

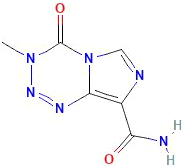

Mechanistically, temozolomide causes a reduction in O^6^‐methylguanine methyltransferase (MGMT), which is required for DNA repair (Figure 2A). This reduction in the enzyme results in an increased level of O^6^‐methylguanine in DNA, leading to a higher cytotoxic effect.^171^, ^172^ A phase III clinical trial in adults reported a higher survival rate of around 11% for radiotherapy combined with temozolomide when compared to radiotherapy alone.^173^ Because of its good tolerance, temozolomide is prescribed for most cases of glioblastoma in patients.^174^ Unfortunately, this drug's utilization post‐radiotherapy has not shown benefit in pediatric DIPG cases.^175^, ^176^, ^177^, ^178^ A comprehensive review compiling different trial studies pointed to no substantial difference in overall survival compared to the control treated with only radiotherapy.^132^

One of the critical reasons for the ineffectiveness of this drug against DIPG is the BBB.^179^ Despite the small size of temozolomide (194 Da) and associated lipophilicity, the detected concentration of the drug in brain tumor tissue is only about 17.8% of the plasma level with mean area under‐concentration‐time curve (AUC) for plasma level 17.1 and 2.7 μg/mL × h for brain.^180^ The differential integrity of the BBB (different parts of the brain) was observed for the selective permeability of temozolomide in the pontine, the cortex, and CSF, suggesting a location‐based phenotype for the BBB.^181^, ^182^ A study by Ostermann et al. showed CSF levels of temozolomide in patients with newly diagnosed recurrent malignant gliomas were consistently in the 20% of plasma level range but could get up to 35% of the plasma levels when co‐administered alongside radiation.^183^ Controlled and targeted radiation can be used for a transient opening and modulation of the BBB neurovascular unit for better drug penetration.^183^ Studies are ongoing to improve BBB penetration for temozolomide by employing techniques like focused ultrasound, regadenoson (a vasodilating process), and nanoparticles to enhance penetration and inhibit transporters.^184^, ^185^, ^186^, ^187^

#### 3.2.4 Bevacizumab

**FIGURE 3:**
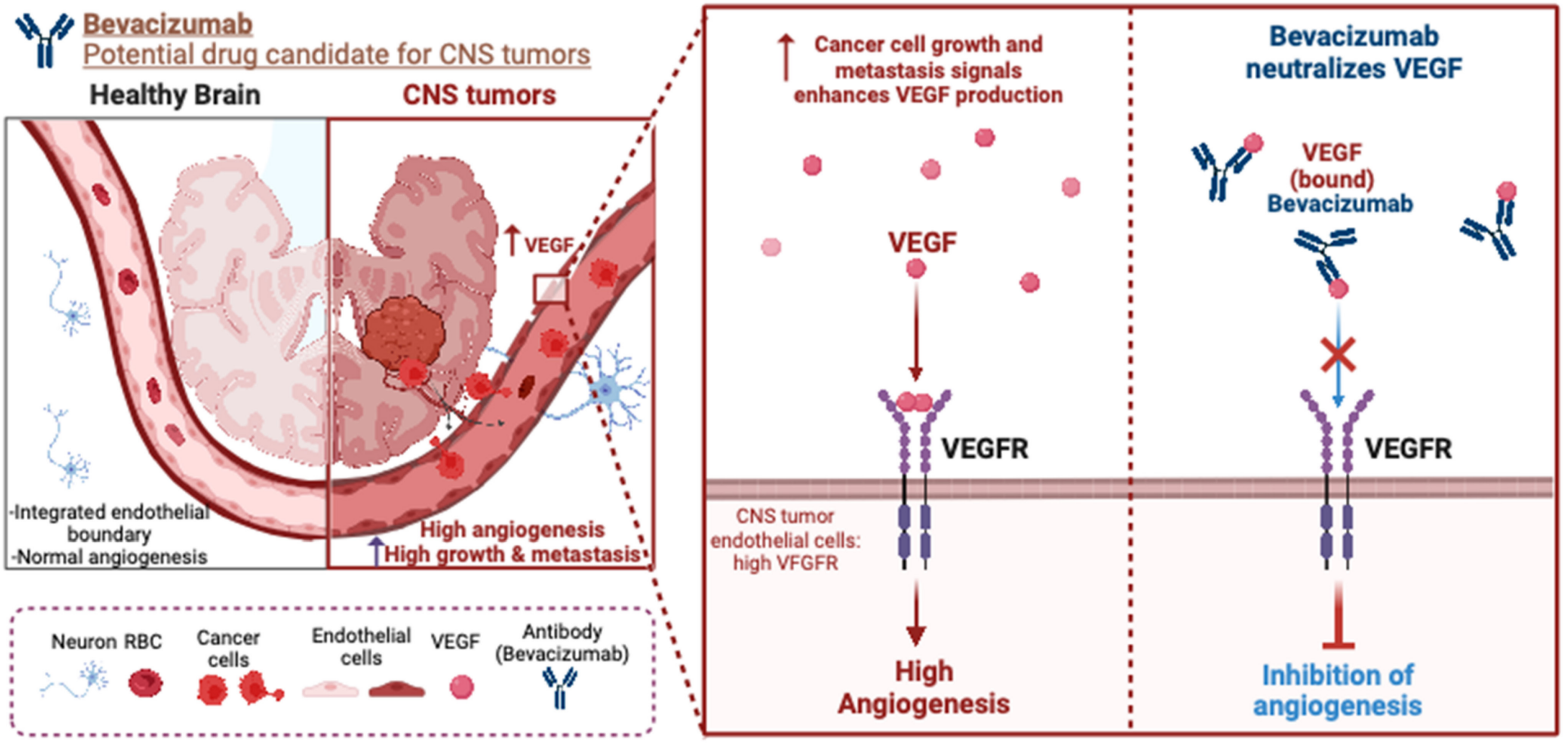
Bevacizumab is a humanized monoclonal antibody that inhibits angiogenesis by neutralizing vascular endothelial growth factor (VEGF), which is upregulated in tumor growth and metastasis of multiple types of CNS tumors. CNS, central nervous system; VEGFR, vascular endothelial growth factor receptor. *Figure was generated utilizing Biorender.com.

Bevacizumab is a recombinant and humanized monoclonal antibody (mAb) with high specificity and affinity for VEGF^188^, ^189^; the mechanism is shown in Figure 3. VEGF is pivotal in tumor growth and metastasis in children.^189^ Prior to the primary tumor resection, children with cancer have increased circulating VEGF levels.^190^, ^191^ Clinical data show overexpression of VEGF‐A and its receptor VEGFR2 in various brain tumors, including DIPG.^189^, ^192^ Bevacizumab appears to be relatively safe for children with primary CNS tumors.^193^ Non‐randomized trials of bevacizumab in children diagnosed with PBT show varying levels of clinical improvements.^158^, ^194^, ^195^ Parekh et al demonstrated a 6‐month progression‐free survival of 38% in patients <21 years of age with WHO grade 3–4 gliomas who receive bevacizumab alone or in combination with CCNU1.^97^ Hummel and colleagues demonstrated that bevacizumab‐based therapies were feasible and safe in HGG and DIPG pediatric patients but did not improve survival in patients with DIPG.^195^ Currently, for DIPG, trials are being conducted with VEGF‐neutralizing mAb.^196^, ^197^, ^198^ A decrease of 65% in tumor size was observed with bevacizumab in combination with temozolomide, but the study only included two patients, raising questions about this combination's clinical benefit.^199^

The VEGF antagonist mAb does not require crossing the BBB as it can directly bind to VEGFR2; however, reaching the distal part of CNS will be more beneficial for treating DIPG.^200^ Currently, there are no clear data on the receptor‐based transcytosis of mAb across the BBB. Since antibodies are relatively large (150 kDa), this will likely hinder mAb's ability to cross the BBB.^201^

### 3.3 Ependymoma

Ependymomas are tumors with a slow proliferating rate. In most cases, fractionated radiotherapy or surgery is the preferred treatment with limited knowledge of the benefits of chemotherapeutic regimes.^202^, ^203^, ^204^ A prominent marker of angiogenesis is the overexpression of VEGF.^205^ Approximately 190 ependymoma‐diagnosed children's cases in the United States are reported yearly with <60% 10‐year or more survival.^43^, ^50^ The standard treatment regime includes near‐total‐resection (NTR) or gross total resection (GTR) followed by fractionated radiotherapy, with an exception in children under 3 years of age.^206^ Two clinical trials have tried to validate this resection methodology and confirmed survival rates ranging from 60% to 80%.^206^, ^207^ Other clinical trials have demonstrated positive outcomes with combined therapy of alkylating agents like carboplatin, ormaplatin, and oxaliplatin with or without cisplatin.^208^, ^209^ The progression‐free survival of children treated with only GTR followed by radiotherapy compared to patients who received chemotherapy and NTR showed a comparable effect of around 58% and 67%, respectively.^208^ In addition to an increased progression‐free survival rate, chemotherapy is also desired in situations where complete resection is impossible. A German HIT‐REZ study enrolled 138 pediatric patients for evaluation of systemic chemotherapy and concluded no advantage of chemotherapy in recurrent ependymoma; however, resection followed by chemotherapy extended the survival rate by more than 1 year.^210^ Another phase II trial for sunitinib enrolled 17 children with DIPG and 13 with ependymoma and found no significant anti‐tumor activity of this drug alone.^110^ As such, multiple trials and research efforts are underway to find a better chemical agent for ependymoma inhibition.^211^

ABC transporters are located in tissues of the intestine, liver, kidneys, heart, lungs, brain, placenta, and testis and are highly expressed in tissue interfaces, specifically blood endothelial interfaces like the BBB.^212^, ^213^ Reports have suggested the invariable occurrence of ABC receptors like multiple drug resistance 1 (MDR1) and CRP in different subtypes of ependymoma.^214^, ^215^ The BBB‐associated ABC transporters play a vital role in drug concentration across the BBB, and their primary function is to extrude both endogenous and exogenous molecules, including drugs.^216^ Thus, a detailed, comprehensive analysis of the BBB on chemotherapy for ependymoma is needed.

#### 3.3.1 5‐azacytidine

5‐azacytidine (AZA) was first discovered in 1960 as a pyrimidine analog with the ability to inhibit DNA methylation.^217^ In an in vitro study, AZA was discovered to stimulate the differentiation of human glioblastoma cells while simultaneously reducing the expression of the G protein‐coupled formylpeptide receptor (FPR), which acts as a mediator in the chemotaxis of phagocytic leukocytes. Additionally, AZA was observed to lower global methylation levels within glioblastoma cells, all the while activating the tumor suppressor.^218^ A study involving rabbits and dogs examined the CSF levels of AZA. It demonstrated that AZA was able to penetrate the CNS through the blood‐CSF barrier, with CSF levels reaching 27% and 58% of the plasma Cmax.^219^ In a pilot clinical trial involving six children with recurrent posterior fossa ependymoma, AZA was administered at doses of 10 mg for 12 consecutive weekly infusions into the fourth ventricle tumor resection cavity. Notably, there were no observed neurological toxicities, and two out of five patients exhibited a decrease in the size of intraventricular lesions.^220^

#### 3.3.2 Pembrolizumab

Pembrolizumab is a humanized monoclonal antibody known for its high affinity for programmed cell death ligand 1 (PD‐L1), which is found on antigen‐presenting cells, including cancer cells. It functions by inhibiting the interaction between PD‐L1 and the programmed cell death‐1 (PD‐1) receptor on cytotoxic T‐lymphocytes, thereby enhancing the T‐cell response against cancer cells.^221^ Notably, PD‐1 and PD‐L1 are highly expressed in supratentorial ependymoma and posterior fossa ependymoma. In these cases, the PD‐L1‐PD‐1 interaction serves to protect the host by restraining hyperactive T‐effector cells. However, disrupting this interaction has shown promise in improving anti‐tumor cytotoxic T‐cell immunity.^222^, ^223^, ^224^ In advanced melanoma patients, treatment with pembrolizumab yielded an overall response rate of 33%. Furthermore, there was a 35% rate of progression‐free survival for 12 months, and the median overall survival reached 23 months.^225^ Pembrolizumab has demonstrated effectiveness against various cancer types, as summarized in a report by the European Medicines Agency.^226^ Currently, an ongoing phase I clinical trial of pembrolizumab (Study ID‐NCT02359565) is investigating its use in pediatric brain tumors, including ependymoma, with a focus on identifying side effects and determining the optimal dosing for patients under 18 years of age. However, despite its effectiveness as an immune checkpoint inhibitor (ICI), the success of pembrolizumab also depends on its ability to cross the BBB. Therefore, a phase II trial (Study ID‐NCT05879120) is underway to explore the role of the BBB in influencing the potency of pembrolizumab in the treatment of recurrent glioblastoma in patients over 18 years of age.

## 4 CONCLUSIONS AND FUTURE PROSPECTIVE

We focused this review on the major types of PBTs, their chemotherapeutic treatment, and the involvement of the BBB. PBTs represent a significant treatment challenge, and the success of even highly potent anti‐PBT drugs can be limited by poor penetration through the BBB. The use of chemotherapy was initially as an adjuvant to surgical and radiotherapy‐based treatments, but significant progress has been made in their usage in pediatric brain cancers. Chemotherapy can potentially become a standard treatment modality, thereby eliminating the need for radiotherapy and its associated long‐term side effects. The failure of conventional anti‐cancer drugs in treating PBTs is potentially due to the restrictive cellular barriers that isolate the CNS, maintain homeostasis, and regulate the passage of molecules across the BBB.

Two promising research pathways dominate efforts to improve CNS drug delivery. One is focused on developing novel systemic drug delivery methods, and the other is on directly modulating the BBB to improve CNS drug bioavailability.^81^, ^82^, ^227^, ^228^, ^229^ PET image‐guided HDAC inhibition (PETobinostat) is a recent drug delivery method that combines convection‐enhanced delivery and image guidance is being applied to increase BBB penetration of panobinostat.^153^

There is a need for a better delivery method to counter the BBB resistance for drugs like nimotuzumab, gefitinib, and erlotinib, which have been shown to have some beneficial outcomes in subsets of DIPG.^230^, ^231^ In this regard, in vitro BBB models can be used to screen anti‐tumor compounds in a timely manner and evaluate the BBB integrity modulation for desired drug penetration before clinical data are available.^232^, ^233^, ^234^, ^235^ More research is needed in this area to optimize the bioavailability of anti‐tumor agents across the BBB and augment PBT therapeutic options.

## AUTHOR CONTRIBUTIONS

**Johid Reza Malik:** Conceptualization (equal); project administration (equal); supervision (equal); validation (equal); visualization (equal); writing – original draft (lead); writing – review and editing (equal). **Anthony T. Podany:** Project administration (equal); visualization (equal); writing – review and editing (equal). **Parvez Khan:** Writing – review and editing (equal). **Christopher L. Shaffer:** Writing – review and editing (equal). **Jawed A. Siddiqui:** Writing – review and editing (equal). **Janina Baranowska‐Kortylewicz:** Writing – original draft (equal); writing – review and editing (equal). **Jennifer Le:** Writing – review and editing (equal). **Courtney V. Fletcher:** Writing – review and editing (equal). **Sadia Afruz Ether:** Writing – review and editing (equal). **Sean N. Avedissian:** Funding acquisition (lead); project administration (equal); supervision (equal); validation (equal); visualization (equal); writing – review and editing (equal).

## FUNDING INFORMATION

We acknowledge support from the following grants from the National Institutes of Health: K23 MH125734 (to SNA) and K23 AI134307 (ATP). The content is solely the responsibility of the authors and does not necessarily represent the official views of the National Institutes of Health.

## CONFLICT OF INTEREST STATEMENT

All authors, no relevant conflicts.
